# Atomistic Insights into the Droplet Size Evolution during Self-Microemulsification

**DOI:** 10.1021/acs.langmuir.1c03099

**Published:** 2022-03-03

**Authors:** Yuequn Fu, Senbo Xiao, Siqi Liu, Yuanhao Chang, Rui Ma, Zhiliang Zhang, Jianying He

**Affiliations:** NTNU Nanomechanical Lab, Norwegian University of Science and Technology (NTNU), Trondheim 7491, Norway

## Abstract

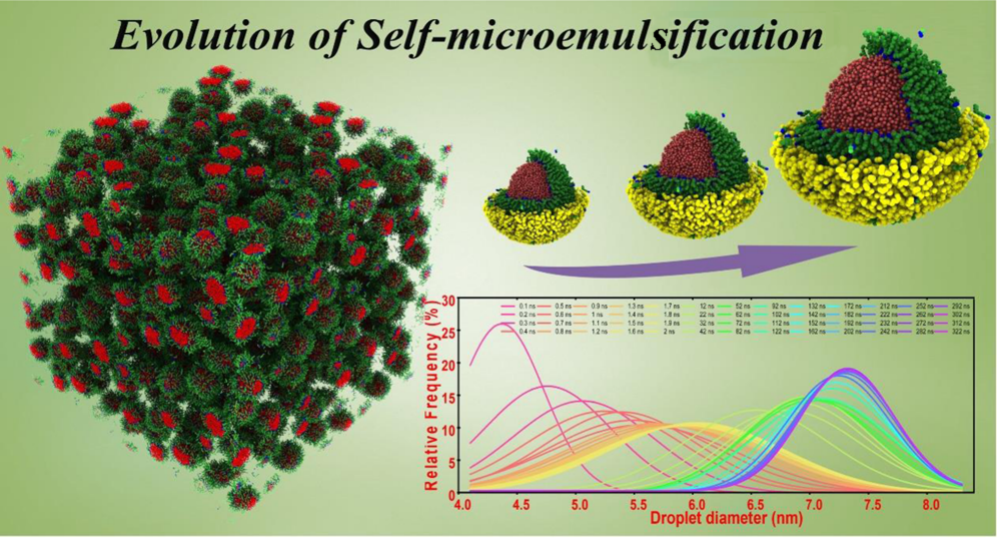

Microemulsions have been attracting great attention for their importance in various fields, including nanomaterial fabrication, food industry, drug delivery, and enhanced oil recovery. Atomistic insights into the self-microemulsifying process and the underlying mechanisms are crucial for the design and tuning of the size of microemulsion droplets toward applications. In this work, coarse-grained models were used to investigate the role that droplet sizes played in the preliminary self-microemulsifying process. Time evolution of liquid mixtures consisting of several hundreds of water/surfactant/oil droplets was resolved in large-scale simulations. By monitoring the size variation of the microemulsion droplets in the self-microemulsifying process, the dynamics of diameter distribution of water/surfactant/oil droplets were studied. The underlying mass transport mechanisms responsible for droplet size evolution and stability were elucidated. Specifically, temperature effects on the droplet size were clarified. This work provides the knowledge of the self-microemulsification of water-in-oil microemulsions at the nanoscale. The results are expected to serve as guidelines for practical strategies for preparing a microemulsion system with desirable droplet sizes and properties.

## 1 Introduction

The interest in microemulsions has been constantly renewed since their discovery in 1943.^1^ Nowadays, microemulsions have found broad applications in many fields, including nanomaterial fabrication,^2,3^ food industry,^4,5^ drug delivery,^6^ and enhanced oil recovery.^7−10^ Microemulsions, as isotropic and transparent liquid mixtures of water, oil, and a surfactant, are thermodynamically stable, which are by definition different from emulsions and nanoemulsions.^11^ Recently, preparing stable microemulsions with a uniform particle size has been shown to be a practical pathway for fabricating functional nanomaterials with desired microstructures.^2,12−14^ Meanwhile, great efforts are still needed for the exploration of the exact controlling parameters of stability, particle size distribution, and morphology of microemulsions.^15^

Microemulsions are sometimes confused with similar systems of nanoemulsions, largely owing to the prefix of “micro-” and “nano-” in the system terminology.^11^ It is known that the droplets in microemulsions, namely, the microemulsified state of a water/surfactant/oil mixed system, are thermodynamically stable, while the ones in nanoemulsions are kinetically stable.^11,16,17^ As such, the droplets in microemulsions are able to maintain their sizes but are subjected to changes in nanoemulsions.

**Figure 1:**
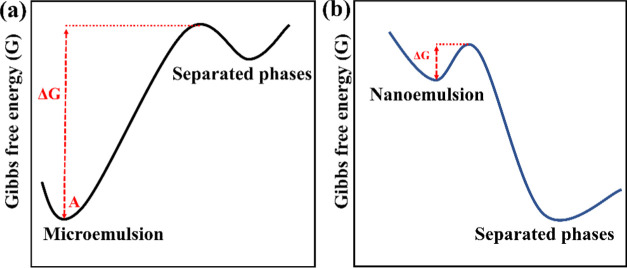
Free energy landscape of a water/surfactant/oil system at different states. (a) Energy difference between microemulsions (marked as state A) and separated phases. (b) Energy difference between nanoemulsions and separated phases.

The difference in Gibbs free energy between the mixed and the phase-separated states of a water/surfactant/oil mixed system can be summarized as eq 1
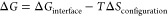
1where Δ*G*_interface_ is the difference in interfacial free energy of different phases and *T*Δ*S*_configuration_ is the difference in configuration entropy of the phases. Compared to the phase-separated state, the Δ*G*_interface_ of mixed states is always positive owing to the positive interfacial tension and increased interface area. The *T*Δ*S*_configuration_ depends on the ways of arrangement of phases within the system, which is negative owing to the higher number of arrangements in the mixture than in the phase-separated state. In addition, there is an optimum curvature or size of droplets determined by the structural and chemical properties of the surfactant molecules, which determines the droplet sizes in microemulsions according to previous studies.^14,18,19^ As such, the Gibbs free energy landscapes separating microemulsions and nanoemulsions from separated phases are drastically different, as depicted in Figure 1. Microemulsions reside at the global minimum, namely, thermodynamically stable, while nanoemulsions reside at the intermediate local minimum, namely, metastable or kinetically stable, of the system Gibbs free energy profile.^20^ As a result, microemulsification of a water/surfactant/oil system is a spontaneous process of energy minimization, while nanoemulsification is an energetically unfavorable procedure. Nevertheless, nanoemulsions are often observed due to their metastable state.

For the evolution of a water/surfactant/oil mixed system, the energy barriers between different states (Figure 1) and mass transport phenomena are two crucial determining factors.^11^ On the one hand, the height of energy barriers determines the level of stability of the current state.^11^ On the other hand, mass transport phenomena cause the microstructural arrangement of small droplets in a water/surfactant/oil system in the process of phase mixing or separation, which is responsible for the transfer and reorganization of molecules in colloidal systems. The mass transport phenomena have been serving as the focus of research interest. Different mechanisms of mass transport, namely, Ostwald ripening, Lifshitz–Slyozov–Wagner theory (LSW theory), and emulsion polymerization, were proposed for explaining the stability of emulsion or nanoemulsion systems,^21−24^ which shed light on the understanding of experimental observations such as flocculation, coalescence, creaming, and so on. Ostwald ripening is a thermodynamically driven process achieved by the fact that molecules energetically favor larger particles rather than smaller ones.^25^ Lifshitz–Slyozov–Wagner theory (LSW theory), as a mathematical performance of Ostwald ripening, indicates that the boundary is among small, shrinking particles and large, growing particles.^26^ The normal type of emulsion polymerization^27^ often happens in an oil-in-water emulsion, which was first explained by Smith–Ewart–Harkins theory.^27,28^ Previous simulations using the dissipative particle dynamics (DPD) method indeed demonstrated obvious Ostwald ripening, the molecular transition from small droplets to large ones, in emulsion systems, which provided strong theoretical evidence at the atomistic scale.^29,30^ Because both the sizes and positions of droplets in emulsions and nanoemulsions change extremely fast, owing to the metastable nature, it is highly challenging to characterize the morphological evolution of microemulsions in experiments. Despite the previous efforts devoted to estimating a single peak droplet size distribution of microemulsions using light scattering measurements,^15,31,32^ the fast dynamical process of formation of stable droplets is still underexplored. Uncertainty in the atomistic mechanisms governing the self-microemulsifying process and the variation of droplet sizes await better clarification and understanding. Furthermore, impacting factors, such as temperature and the properties of surfactant molecules, on the stability of microemulsions need to be thoroughly studied.^33,34^

Herein, our work aims to explore further atomistic insights into droplet evolution in the preliminary stage of self-microemulsification of a water/surfactant/oil system by molecular dynamics (MD) simulations. Starting from well-mixed systems of water, oil, and surfactant molecules, the fast dynamics of forming stable microemulsion droplets was dissected with atomistic resolution. The variation of the morphology and droplet size distribution in the colloidal system was monitored and analyzed for revealing the fundamentals of controlling droplet sizes during self-microemulsification. The results thus provide theoretical references for microemulsion fabrication in relevant studies and applications.

## 2 Models and Methods

### 2.1 Atomistic Modeling

**Figure 2:**
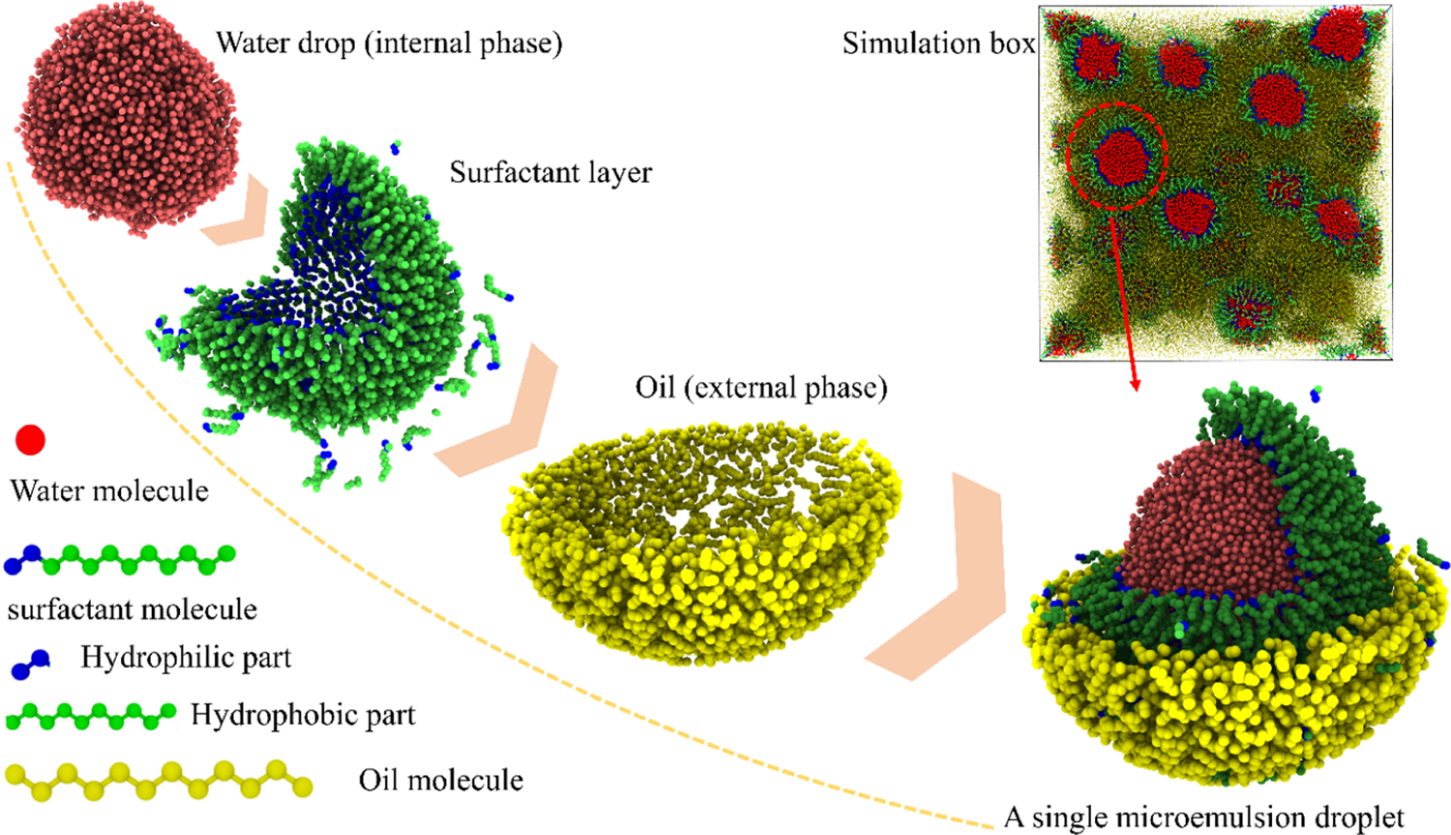
Modeling of surfactant-coated water droplets in the oil phase. The structures of the three molecular types are colored differently for visualization. A representative droplet is dissected, showing the internal detailed layered structure.

Due to the large number of atoms and long simulation running time needed for the formation of a sufficiently large system with a sufficient number of droplets for statistics, coarse-grained modeling was employed in this work. The system used in this study contained three types of molecules, namely, water, surfactant, and oil molecules. The mW model with a classical many-body potential was adopted for the water molecules.^35^ This water model uses a three-body Stillinger–Weber potential to capture nonbonding interactions, featuring hydrogen bonds, among neighboring water molecules. Herein, water was considered as the disperse phase in the final water-in-oil (W/O) microemulsion systems. For the continuous phase, dodecane molecules were chosen to model the oil. Linear diblock oligomer molecules were used as surfactant molecules, following the modeling strategy in previous studies.^36^ Briefly, the CH_2_ groups in the oil and surfactant molecules were treated as one united atom, as shown in Figure 2. The transferable potentials for phase equilibria (TraPPE)^37^ were used for both the oil and surfactant molecules. The amphiphilic property of surfactant molecules was specifically represented by two predetermined lengths of hydrophilic (labeled L) and hydrophobic (labeled B) parts, capturing the molecular properties of linear diblock oligomer surfactants. Besides water–water interactions, the other nonbonded atomic interaction parameters are treated by the Lennard-Jones potential (eq 2) with a cutoff distance of 9.0 Å, with detailed values given in Table 1. This atomic parameter set was borrowed from previous studies^36−41^ and features the formerly used minimalist model.^42^ The parameters of bonded interactions including bonds, angles, and dihedrals in the oil and surfactant molecules were borrowed directly from the TraPPE force field used in alkanes.^43^ The system contained 96 000 water molecules, 16 000 surfactant molecules, and 480 000 oil molecules, with a total of 864 000 untied atoms in an evenly mixed initial state, as shown in Figure S1. The simulation box was set periodic, with a volume of 312.8 Å × 312.8 Å × 312.8 Å in equilibrium, as shown in Figure 2.
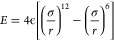
2

**Table 1:** Detailed Interaction Parameters, Energy Depth (ϵ) and van der Waals Radius (σ), of the LJ Potential between Different Atoms

	W:W	W:L	W:B	W:O	L:L	L:B	L:O	B:B	B:O	O:O
ϵ (kcal/mol)	SW^35^	0.602	0.119	0.119	0.602	0.091	0.091	0.091	0.091	0.091
σ (Å)	SW^35^	3.558	3.558	3.558	3.558	3.95	3.95	3.95	3.95	3.95

### 2.2 Molecular Dynamics (MD) Simulations

The Large-scale Atomic/Molecular Massively Parallel Simulator (LAMMPS) package^44^ was utilized to perform all of the simulations. Before MD simulations, the system was energy minimized using the steepest descent algorithm. All MD simulations were carried out under the NPT ensemble, with a system temperature of 298 K coupled by the Nose–Hoover thermostat^45^ and a pressure of 1 bar coupled by the Parrinello–Rahman barostat.^46^ The temperature and pressure coupling constants were 1 and 10 ps, respectively. The simulation time step was 10 fs. Larger time steps enabled a longer simulation time for forming such a big simulation system. The coarse-grained model enabled fast dynamics of the simulation system, which was suitable for this study.^47^ There was a vacuum space in the initial system, which was eliminated in the early steps of the simulation. In the first 2 ns, the trajectories of all atoms were recorded per 0.1 ns, and then per 10 ns, to capture the fast formation and the morphology dynamics of microemulsion droplets.

**Figure 3:**
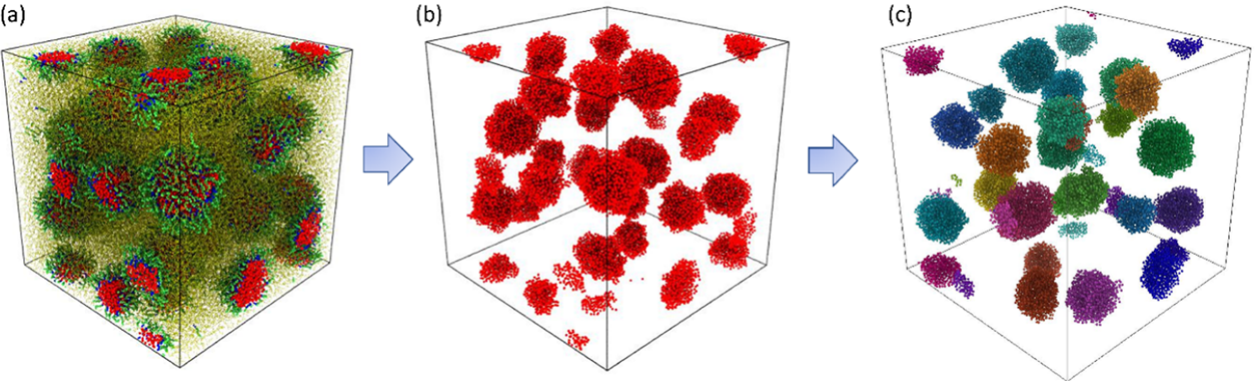
Characterization of droplet sizes in the simulation system. (a) View of the whole simulation box after the formation of droplets. (b) Water cluster by a neighboring search with a cutoff distance of 3.2 Å. The oil molecules and surfactant molecules are not shown for the better visualization of the individual droplets. (c) Individual droplets, represented by the water core, are labeled in different colors. The number of water molecules in every water cluster was counted for evaluating the droplet diameter and surface area.

With the ratio of water and oil molecules modeled in the system, the resulting droplets had a water core and surfactant shell dispersed in a continuous oil phase. To characterize the diameters of the resulting droplets, all of the droplets in the system were identified by a neighboring cluster search with a cutoff distance of 3.2 Å, as shown in Figure 3. The work of cluster analysis was done using the software OVITO.^48^ The function of the cluster was achieved by a modifier that can decompose the particles into disconnected groups (so-called clusters) based on the selected neighboring criterion. The neighboring criterion can be distance-based (cutoff range) or topology-based (bond network).^48^ Here, it is distance-based (a cutoff range of 3.2 Å). Each water cluster, with the size approximately taken as the size of the whole surfactant-coated droplet, was then approximatively considered as a sphere. By counting the number of water molecules (*N*_W_) and the volume of a single water molecule (ρ, 29.9 Å^3^) in each water cluster, the diameter (*D*) and surface area (*A*) of each droplet were estimated using the following eqs 3 and 4
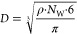
3
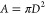
4

The diameters of all of the droplets in the system were monitored and collected throughout the simulations. For clarifying the effect of temperature on the size of droplets, the temperature range of 280–400 K (6.85–126.85 °C) was chosen to carry out the simulations, with the results for detailed comparison. Temperature is an essential factor that affects the evaluation of the droplet. Due to the complexity and the content ratio of the component in microemulsions, the boiling point of the microemulsions is not constant under atmospheric pressure. Meanwhile, the boiling point of the system also depends on the type of water model.^49^

## 3 Results and Discussion

### 3.1 Morphology and Droplet Size Distribution in the Water/Surfactant/Oil System during Self-Microemulsification

**Figure 4:**
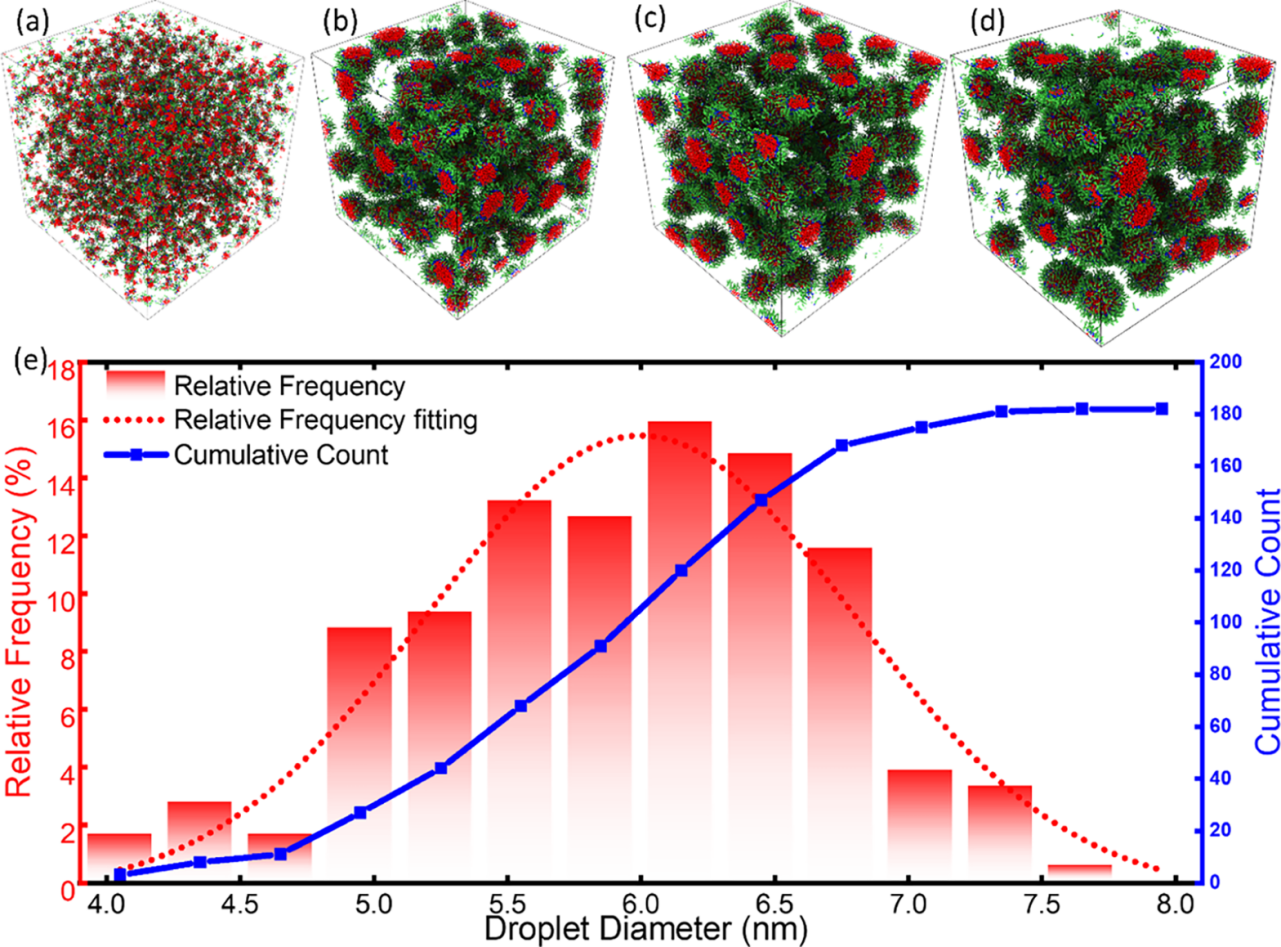
Droplet sizes in the system during the simulation. (a–d) System snapshots of droplet sizes at different times of 0.1, 10, 150, and 300 ns, respectively. Oil molecules are hidden for better visualization of the droplets in the system. (e) Droplet size distribution in the early state of simulation (at the time of 1.8 ns). The distribution of the diameters is shown as the relative frequency. The cumulative count indicates the total 180 droplets in the system. The normal distribution of droplet sizes observed in experiments is shown as a dashed line for comparison.

The initial evenly mixed state of water, oil, and surfactant molecules (Figure S1) enabled a fast formation of small droplets. For a practical emulsifying process, the initial free energy of the system should be the red area shown in Figure S2. Mixing or shearing the components is mostly a necessary step to prepare a microemulsion.^20^ In this work, normal mixing was considered as an essential step to prepare a microemulsion to perform a complete self-microemulsification process. Such a step increased the free energy of the system and offered the initial driving force for the automatic formation of small droplets in the preliminary stage of self-microemulsification. After a short time, a colloid dispersion system with many nanoscale spherical droplets was quickly achieved in the early stage, as shown in Figure 4a. These early-formed small droplets were comprised of a water core and a surfactant shell and were dispersed within an oil-continuous medium, which followed the previous theoretical prediction of “droplet microemulsion” or “swollen micelles”.^19^ The surfactant molecules swiftly assembled at the oil–water interface. The hydrophilic heads of the surfactants were oriented toward the water cores of the droplets due to the strong interatomic attraction (Table 1), while the hydrophobic tails stretched into the surrounding oil-continuous phase. As such, the surfactant molecules were arranged in an orderly manner at the oil–water interface, forming spherical surfactant shells, as shown in Figure 2. Such a core–shell structure of the droplets greatly reduced the interfacial tension of the curved interface between the water and oil phase,^50−52^ which optimized the overall energy of the whole system.

**Figure 5:**
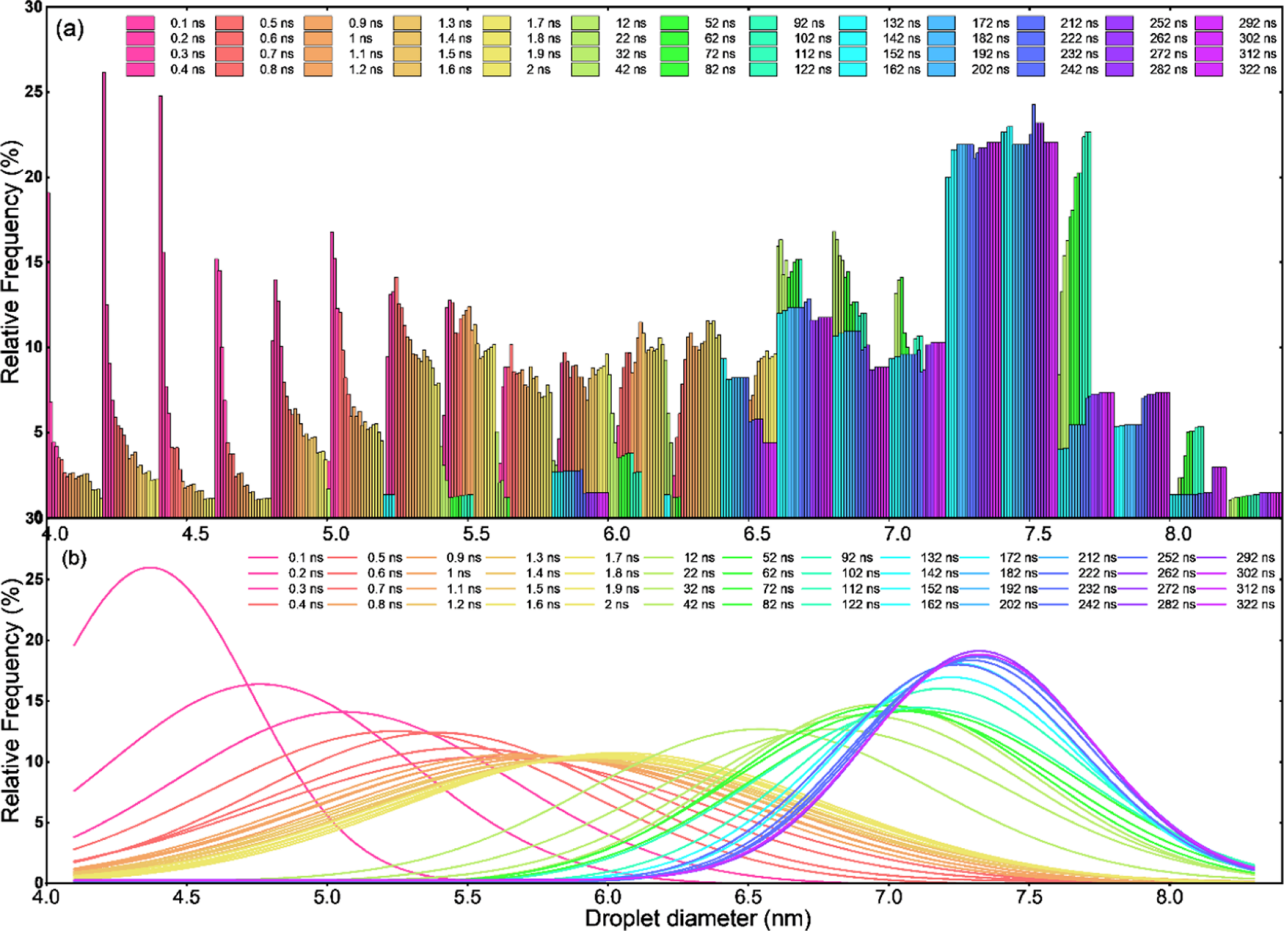
Droplet size distribution in the system during the simulation. (a) Histograms of relative frequency against the diameter size of the droplets at different simulation times. (b) Approximation of the droplet size distribution by Gaussian fitting of the histograms shown in panel (a) for better visualization of the droplet evolution during the simulation.

**Figure 6:**
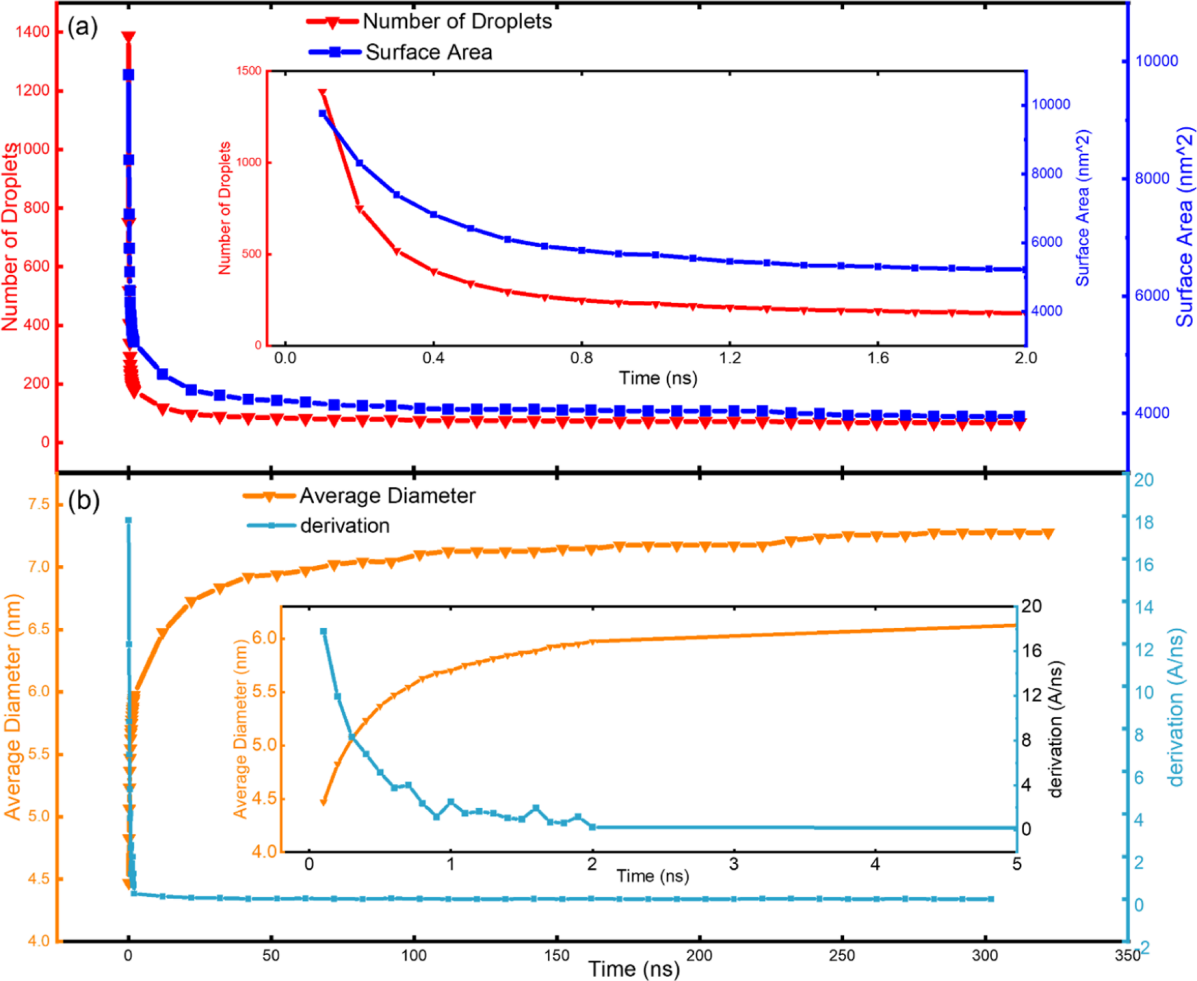
Droplet number and size variation and the resulting changes in the interface surface area. (a) Total number of droplets during the simulation and the resulting total interface surface area in the system. The drastic changes in the droplet number and surface area in the first 2 ns of the simulation are shown in the inset. (b) Average diameter of all of the droplets in the system and its derivation during the simulation. The drastic changes in the average diameter of the droplets and its derivation in the first 5 ns of the simulation are shown in the inset.

Due to the chosen potentials and the water–oil–surfactant molecular ratio, the system progressed extremely fast into a water-in-oil (W/O) dispersion mixture. As shown in Figure 4a, a great number of small droplets formed quickly and spontaneously in a period of 0.1 ns, meeting the purpose of the modeling strategy. The small droplets further aggregated into big ones during the ongoing simulation, mainly by droplet coalescence, as shown in Figure 4b,c. The decrease in the number of droplets also led to a reduction of the oil–water interface area and further lowered system energy. The increasing droplet sizes gradually reached a stable state, especially after a simulation time of 300 ns, as shown in Figure 4d. Such a phenomenon was also explored by energy evolution, as shown in Figures S3 and S4. After 120 ns, the value of potential energy and total energy mostly maintained a constant number, which means that the system reached an equilibrium state. Finally, the average diameter of the droplet remained constant and the size of the droplets became more uniform, as shown in Figures 5 and 6. And as such, at the end of the simulation, the droplets are, or very close to, microemulsions. According to the thermostability and size uniformity of the droplets,^11^ this model can be considered as a microemulsion system.

### 3.2 Evolution of the Size Distribution of Droplets

The droplet sizes were not uniform starting from the beginning of the simulation. For the size distribution of droplets captured at the time of 1.8 ns, for example shown in Figure 4e, the diameter of the droplets ranged from 4.0 to 8.0 nm, falling in the size range (1–100 nm) of typical microemulsion droplets observed in previous studies.^11^ It should be noted that the diameters of the droplets determined here are the diameters of the water core, which are the approximation of the whole surfactant-coated droplets. The peak value of the droplet diameter distribution at this early state was around 6.0 nm, with the most populated sizes in the range of 5.0–7.0 nm. Given that the droplets were actively evolving at this early stage, the distribution of the droplet sizes seemed slightly left-skewed and deviated from a normal distribution commonly observed in experiments.^53,54^ It is worth noting that there were still free water molecules not fully coated by surfactant molecules diffusing in the oil phase at this state, which was also contributing to the increasing sizes of droplets besides the droplet coalescence effect in the ongoing simulations. The droplet size distribution in the system was monitored during the whole simulation for further analysis.

The size of droplets increased with simulation time in this preliminary stage of self-microemulsification until the system reached the equilibrium state of microemulsion. The sizes of all droplets in the simulation system were determined following eqs 3 and 4 during the simulation from 0.1 to 322 ns. From the droplet size distributions at different simulation times shown in Figure 5a and the evolution of the droplets shown in the Supporting Video (self-emulsifying process), the sizes of the droplets increased drastically in the first 50 ns of the simulation and stabilized in the second half of the simulation. Specifically, the size distribution of droplets at the beginning of the simulation was narrow with the peak value below 4.5 nm, as shown in Figure 5b. Because of the fast droplet coalescence and uptake of free water molecules, the distribution of the droplet sizes quickly widened covering a size range of 4–8 nm in the first 2 ns, with the peak value shifted to around 6 nm (yellow-colored distribution, Figure 5b). Starting from 100 ns until the end of the simulation, the distribution of droplet sizes became narrow again, reaching a stable peak value at around 7.5 nm (purple-colored distribution, Figure 5b). It is worth noting that the evolution of the droplet sizes and stabilization of the size distribution at the beginning of the simulation were quick. The coarse-grained modeling enables a fast simulation. But the models do not affect the speed of the evolution of droplets. In this work, coarse-grained modeling just not only saves the computer hours spent in the simulations due to the larger time step of 10 fs but also has a smoother energy landscape and fast dynamics.^47^ So, it is beneficial for this study. The long-term stabilization of droplet size distribution in the second half of the simulation suggested that the system reached an equilibrium state with long-term stable microemulsion droplets resting in the energy state A in Figure 1a. The total system potential energy also showed a plateau in the second half of the simulation as shown in Figures S3 and S4, clearly indicating that the system was in equilibrium. External energy is thus needed to further disturb the stability of the droplets and bring the system to the phase-separated state.

The evolution of the droplet number and sizes directly resulted in the changes of the interface surface area in the water/surfactant/oil colloidal systems. As depicted in Figure 6a, the drastic decrease of the droplet number at the beginning of the simulation in the system, namely, droplet coalescence and the increase of droplet average sizes (Figure 6b), was accompanied by an extremely fast decrease in the interface surface area. The decrease in the interface surface area was greatly favorable to the lowering of the total system energy (Figures S3 and S4) and the stabilization of the droplets. As the droplets gradually stabilized after the early stage of the simulation, the average diameter of the droplet remained constant in the system, showing almost negligible deviation in the average diameter of the droplets (Figures 6b and S5). To better understand this section, Figure S6 is plotted to show more details about analyzing the data.

### 3.3 Temperature Effect on Droplet Sizes

**Figure 7:**
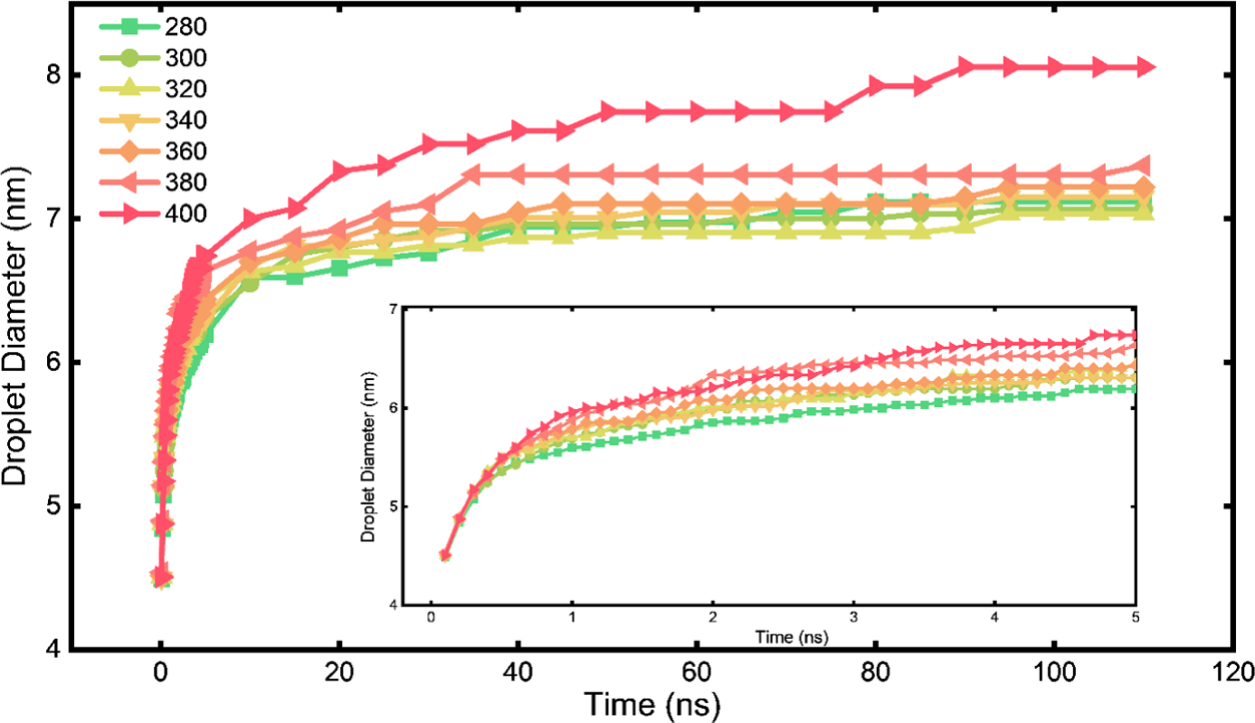
Impact of temperature on droplet evolution and the final sizes. The average droplet sizes during simulations from 280 to 400 K are colored differently in the figure.

It is known that temperature has a significant effect on the microemulsion system.^55,56^ Here, the temperature was also found to have a great impact on the droplet size evolution. Droplet evolutions under different temperatures were compared, as shown in Figure 7. Under seven temperatures of 280, 300, 320, 340, 360, 380, and 400 K, the average droplet diameters all featured a steep increase at the beginning of the simulations and stabilized in the final equilibrium state (Figure 7). Generally, higher temperatures led to a faster increase in the average droplet diameter in the initial stage of the simulations. As the inset in Figure 7 shows, larger droplets were observed with the higher temperature at the same simulation time, which could be attributed to the higher possibility of droplet collision and coalescence and faster molecular dynamics at the higher temperature. As such, the results suggested that a higher temperature can accelerate the spontaneous microemulsifying process. A higher temperature also led to averagely bigger droplets, as indicated by the final average droplet sizes at the end of the simulation shown in Figure 7. It should be noted that the high temperatures, especially 380 and 400 K, were used here only to demonstrate the obvious temperature effect, which is higher than the phase separation temperature and boiling point of some microemulsion systems. Despite the short simulation time, the droplet size observed in the simulation at 400 K was much larger than those at the lower temperatures. It could be speculated that a phase separation could be expected with a longer time. In addition, the relationship between the droplet size and the temperature is explored in Figure S7. The result, shown in Figure S7b, clearly indicated the presence of an optimum temperature at which the size of the droplets formed was at its minimum. Away from the optimum temperature, any temperature change will increase the droplet size, similar to the result from an experimental study.^57^

### 3.4 Mass Transport Mechanisms in Self-Microemulsification

**Figure 8:**
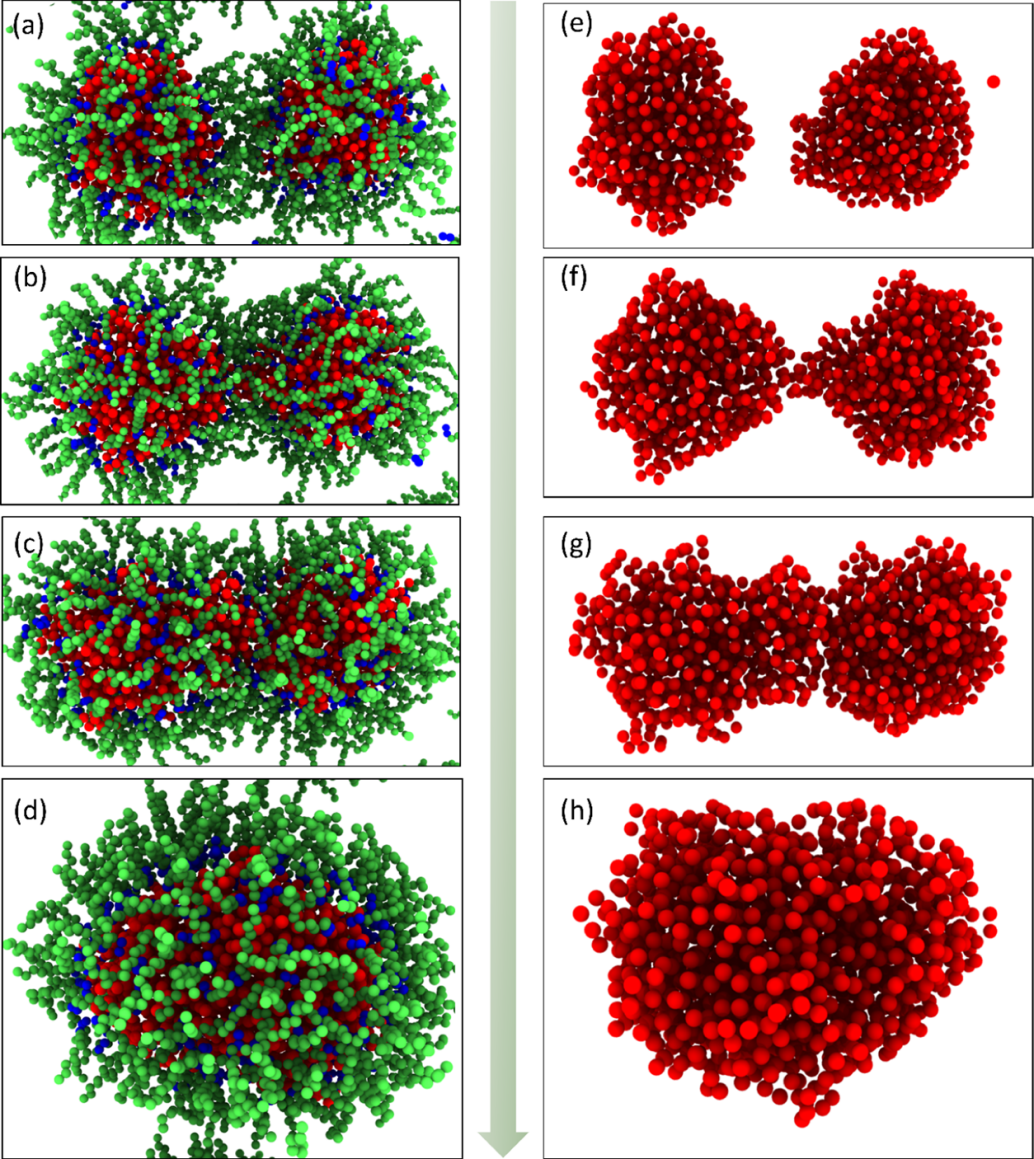
Coalescence of two droplets. (a–d) Whole coalescence shown with the surfactant shell of the two droplets. (e–h) Coalescence process shown by only the water cores of the two droplets.

**Figure 9:**
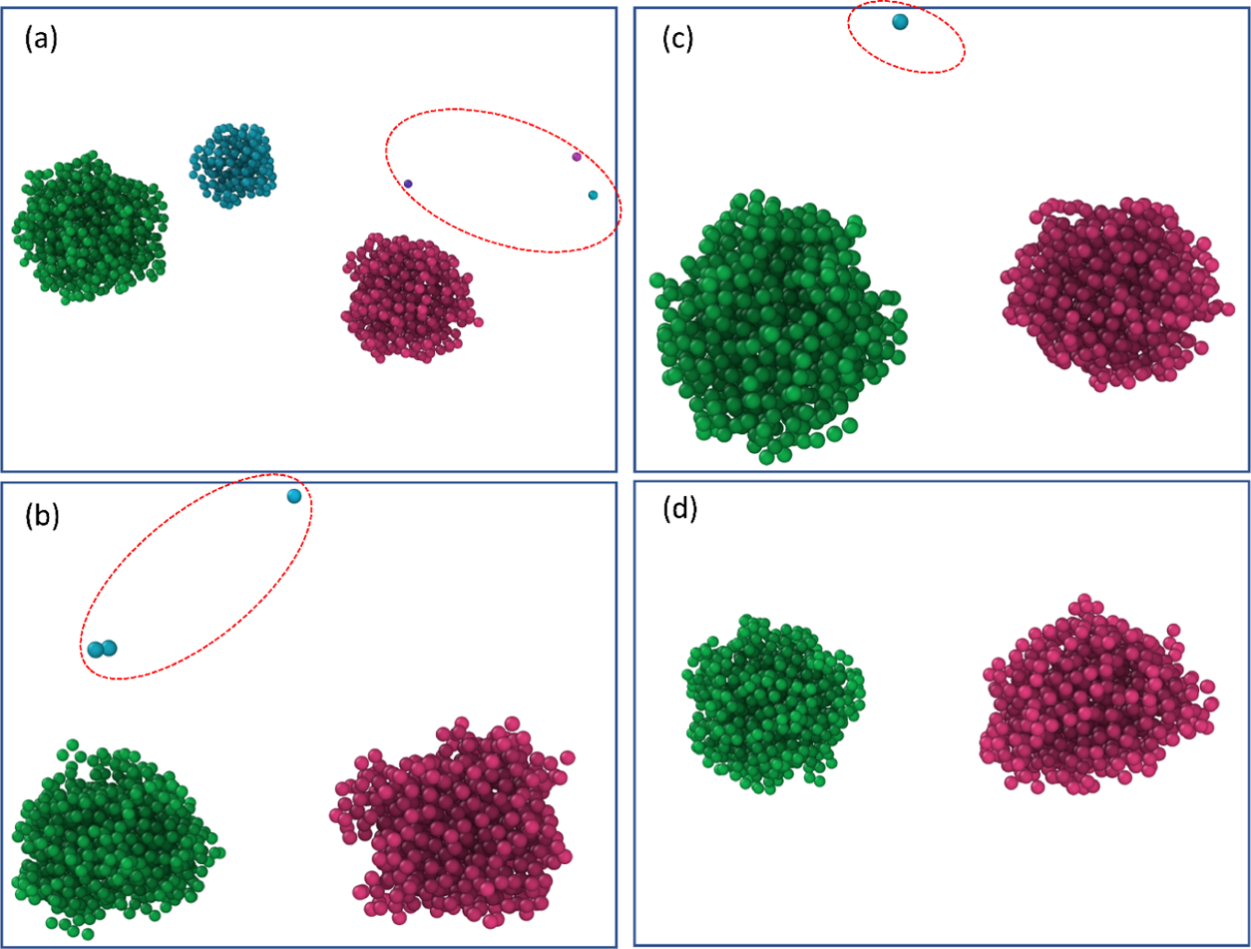
Ostwald ripening among droplets in the system. Water cores of different droplets are shown with different colors. The water molecules detached from the droplets and diffused in the oil phase are highlighted by circles. (a) Snapshot of the system at 0.2 ns, (b) snapshot of the system at 0.5 ns, (c) snapshot of the system at 1 ns, and (d) snapshot of the system at 1.8 ns.

There were two possible modes of increase in the average sizes of the droplets in the system, namely, droplet coalescence and Ostwald ripening between droplets, as discussed in the previous sections. To further reveal the dynamics of droplet coalescence, a smaller system containing only two droplets was built and subjected to simulations with the same controlling parameters, and the results are shown in Figure 8. A coalescence event started with the merging of the hydrophilic tails of the surfactants from two droplets (Figure 8a). Strikingly, a water bridge was gradually formed and enlarged after the merging of the surfactant shell, as shown in Figure 8b,c,f,g and the Supporting Video (aggregating process). Afterward, the two droplets became one large droplet with a spherical shape (Figure 8d,h). In this process, the merging of the surfactant shell was a random and rate-limiting step, while the formation of the water bridge and final coalescence were highly efficient. Ostwald ripening was observed among droplets during the whole course of simulations. As shown in Figure 9, water molecules in small droplets were more likely to detach from the original droplet into the oil phase, owing to the high Laplace pressure that resulted from the big surface curvature. The detached water molecules can then freely diffuse in the system and finally be captured by other droplets. As a net result, water molecules diffused from smaller droplets to bigger ones, yielding the same Ostwald ripening process observed in other previous studies.^22,58^

The two mass transport mechanisms contributed to the whole self-microemulsifying process, with different probabilities at different stages. Namely, droplet coalescence dominated the early stage of droplet size evolution, which greatly drove the drastic increase of droplet sizes in the system. In the simulations carried out in this work, the period of active droplet coalescence was largely the first 5 ns. In comparison, Ostwald ripening occurred in the whole process of the simulation and was the dominating mass transport mechanism among droplets in the system close to the equilibrium state. It is important to mention that with the gradual growth of big droplets and shrinkage of small ones, the surfactant molecule shell rearranged to accommodate the change of the water core volume. Specifically, the surfactant shell became loosened on larger droplets while tightened and compacted on smaller ones, which slowed down mass transport and eliminated the variation of the droplet sizes.

## 4 Conclusions

In summary, a series of molecular dynamics simulations were carried out to explore the droplet size evolution and mass transport mechanisms in the primary stage of the self-microemulsifying process. Thanks to the coarse-grained models chosen, the simulations were able to reveal the whole process of self-microemulsification, reaching stable microemulsion systems. Surfactant-coated droplets were found to go through two distinguished steps before the whole colloidal system became a stable microemulsion, namely, a rapid droplet size growth step at the beginning of mixing via active droplet coalescence and a slow evolving step of droplet size stabilization by Ostwald ripening. The fast droplet coalescence led to a wide distribution of the droplet sizes in the system, while Ostwald ripening eliminated smaller droplets in the system and narrowed the droplet size distribution in the final stage of microemulsification. Furthermore, the temperature can impact the speed of evaluation and final morphology of self-microemulsification. Higher temperatures accelerated the whole process and resulted in larger droplet sizes. It should be noted that the key parameters, such as the length of the hydrophobic chain and the types of hydrophilic head groups, influence the droplet size evolution and are worth further study. The results provide a better understanding of droplet size variation during self-microemulsification and can act as guidelines for the design and fabrication of microemulsions with desired sizes and configurations.

## Supporting Information Available

General dynamic view of evolution of the colloid system from the mixture state to the microemulsion state (self-emulsifying process) (MP4)
Process of two droplet aggregating slowly with each other (aggregating process) (MP4)
Schematic of the evenly mixed initial state of the water/surfactant/oil system (Figure S1); system’s Gibbs free energy at different states (Figure S2); variation of potential and total energy of the system with time (Figure S3); potential energy–time and total energy–time curves of the system (Figure S4); diameter values of the first 30 microemulsion droplets (Figure S5); droplet size distribution and its components (Figure S6); average droplet diameter–temperature curves at different times (Figure S7) (PDF)
