# Supplementary material for: Atomistic Insights into the Droplet Size Evolution during Self-Microemulsification
Source: Langmuir. 2022 Mar 3;38(10):3129–38. doi: 10.1021/acs.langmuir.1c03099 (PMC8928481; doi:10.1021/acs.langmuir.1c03099)
Supplement: Supplementary file 1 — la1c03099_si_001.pdf [file la1c03099_si_001.pdf]

Supporting Information

Atomistic insights into the droplet size evolution during self-microemulsifiction

Yuequn Fu,[a] Senbo Xiao,*[a] Siqi Liu,[a] Yuanhao Chang,[a] Rui Ma,[a] Zhiliang Zhang[a] and Jianying He*[a]

[a]NTNU Nanomechanical Lab, Norwegian University of Science and Technology (NTNU), Trondheim 7491, Norway

*Corresponding author.

E-mail addresses: senbo.xiao@ntnu.no (S. Xiao) and jianying.he@ntnu.no (J. He).

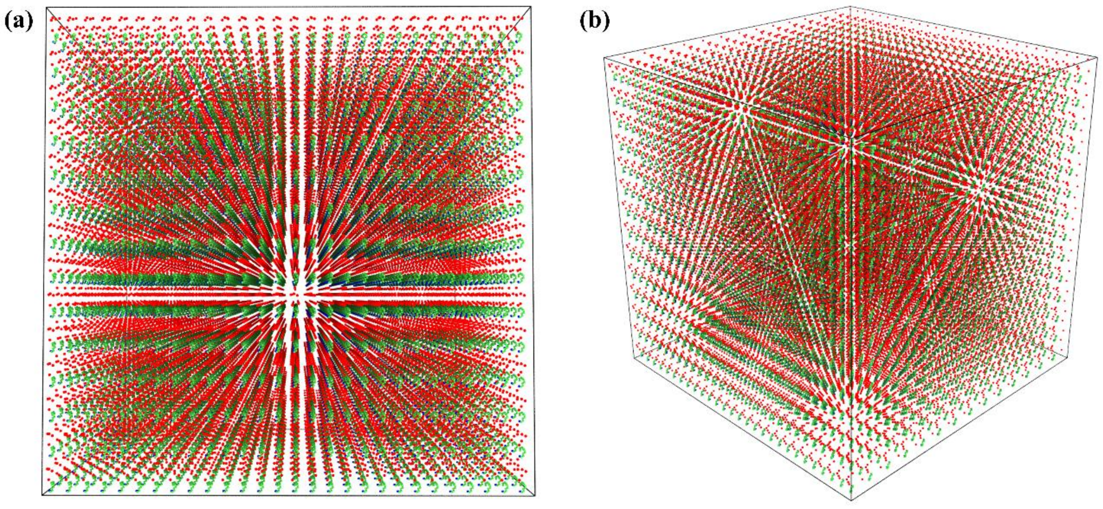

Figure S1. Schematic of evenly mixed initial state of water/surfactant/oil system. (a) Front view of the simulation box, (b) side view of the simulation box.

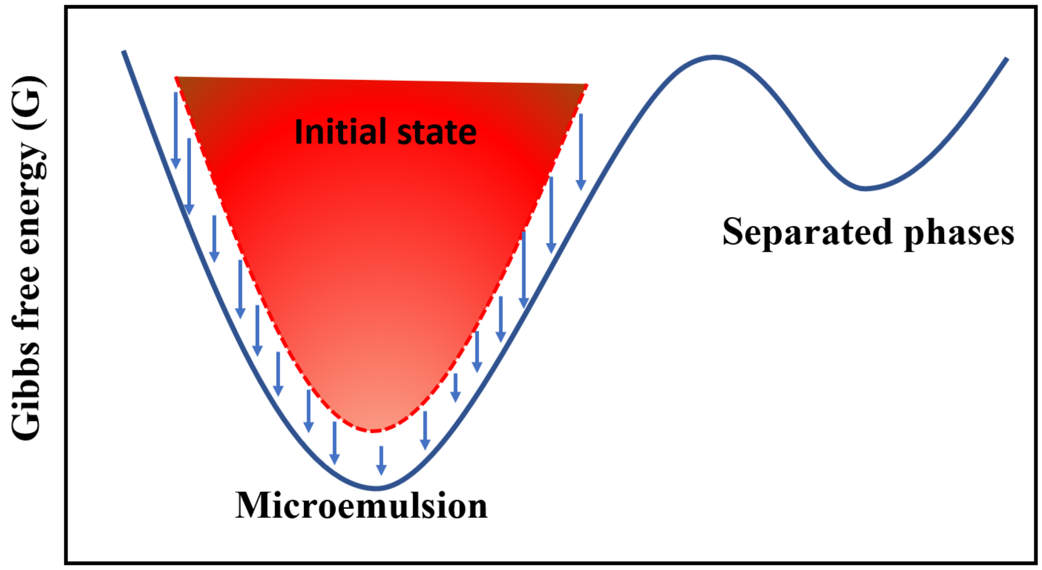

Figure S2. The system's Gibbs free energy at different states.

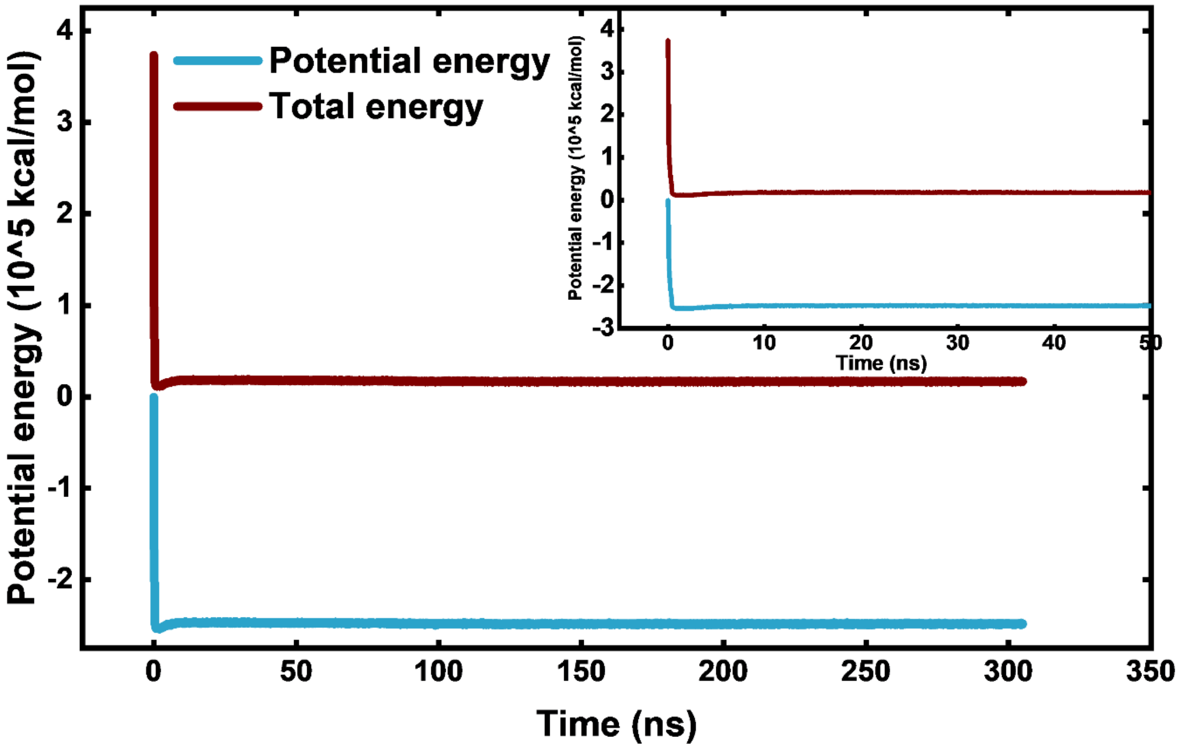

Figure S3. The variation of potential and total energy of system with time.

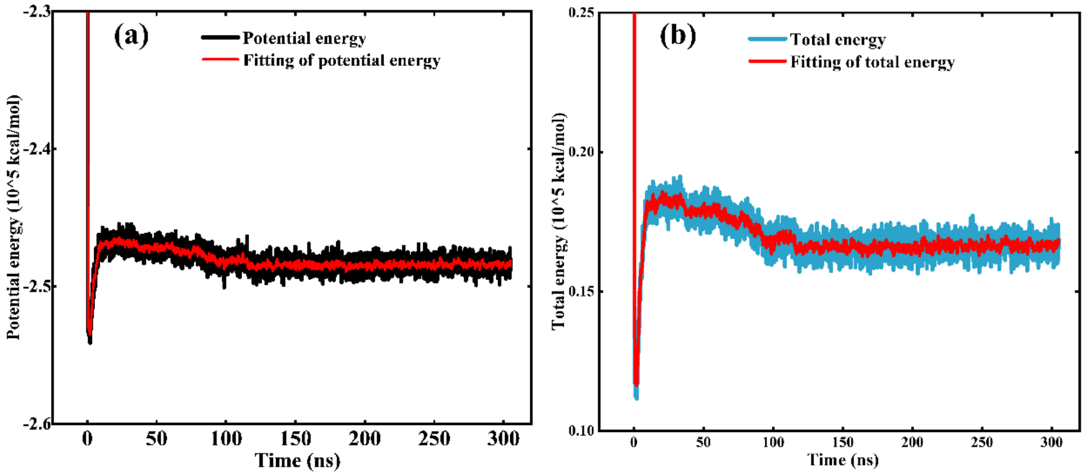

Figure S4. The potential energy-time and total energy-time curves of the system. (a) The evolution of system's potential energy, (b) The evolution of system's total energy.

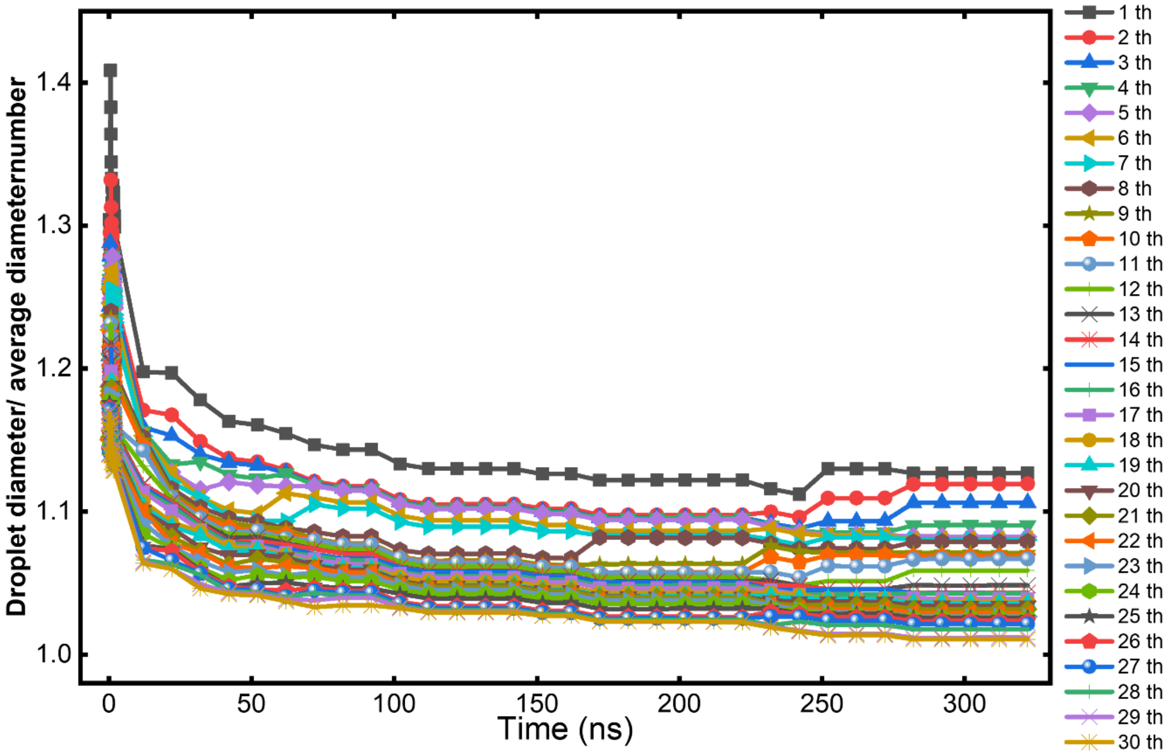

Figure S5. The diameter values of the first thirty microemulsion droplets (from maximum to minimum of droplets diameter) in the microemulsion system were divided by their average value of diameter of overall system in same moment.

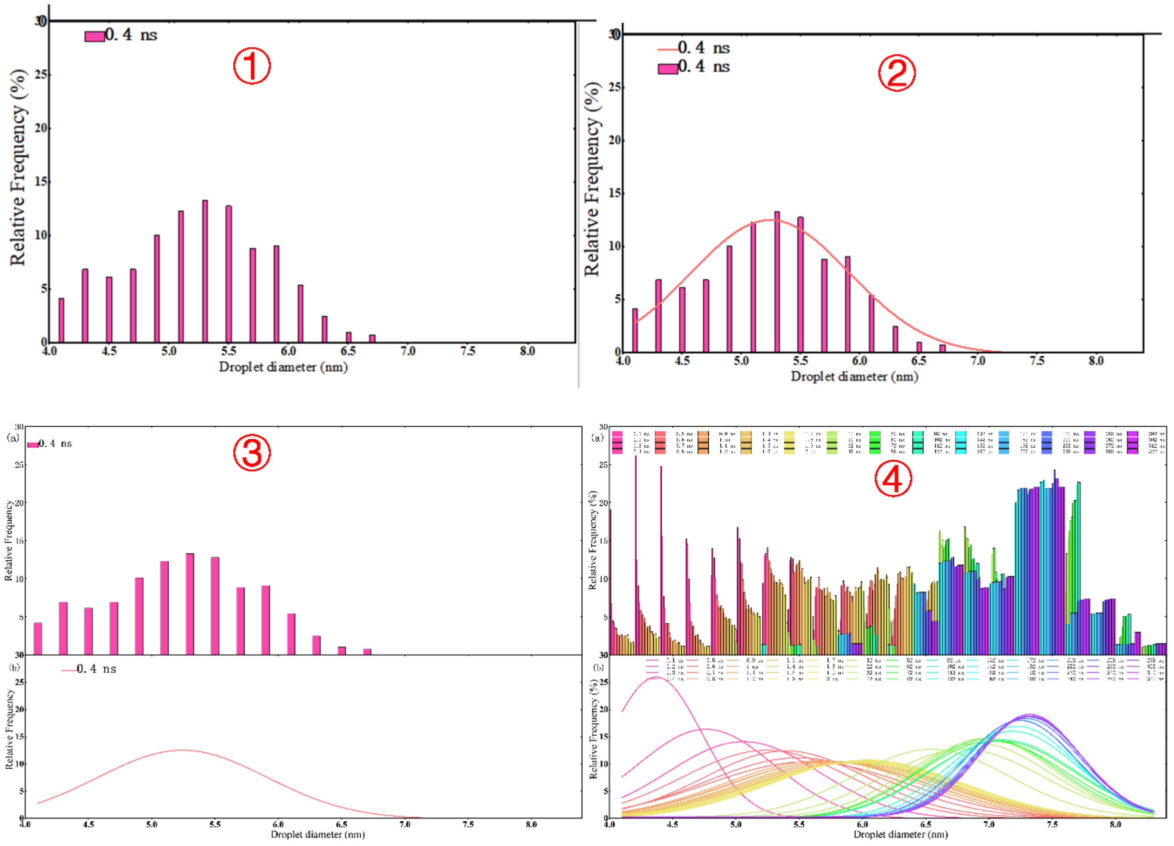

Figure S6. The droplet size distribution and its components.

Figure S6 was plotted to describe how to organize Figure 5 and its components. For example, the system at a specific time of 0.4 ns was chosen to be used. The histogram of droplet size distribution at time of 0.4 ns was collected in Figure S6 ①. With gaussian fitting method, the red curve was used to describe the droplet size distribution, as shown in Figure S6 ②. In order to avoid the overlap between the histogram and curve, they are separated into two figures, as shown in Figure S6 ③. Following the same way, we collected all data at various time from 0.1 to 322 ns into Figure S6 ④.

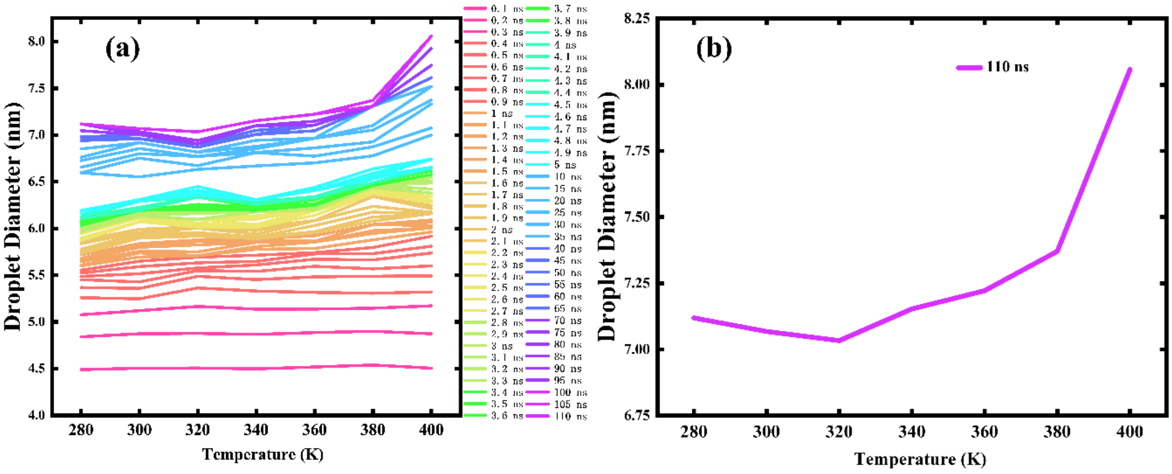

Figure S7. (a) the average droplet diameter-temperature curves at different time. (b) one droplet diameter-temperature curve collected from (a).
